# Acceptorless Dehydrogenative Polymerization

**DOI:** 10.1002/anie.202526144

**Published:** 2026-03-28

**Authors:** Xin Liu, Jessica L. Lathrop, Garret M. Miyake

**Affiliations:** ^1^ Department of Chemistry Colorado State University Center Ave Fort Collins Colorado 80523 USA

**Keywords:** Acceptor‐less dehydrogenative polymerization, Catalysis, Chemically recyclable polymers, Sustainability, Transition‐metal complex

## Abstract

The development of polymerization strategies for enabling the synthesis of polymeric materials with targeted properties remains a central pursuit at the interface of catalysis, polymer chemistry, and materials science. Acceptor‐less dehydrogenative polymerization (ADP) has emerged as an approach for a step‐growth polymerization for the construction of polyesters, polyamides, polyurethanes, and polyureas from unactivated alcohols and amines, liberating molecular hydrogen as the only by‐product. Mechanistically, ADP represents the macromolecular extension of the **x** framework, wherein transition‐metal catalysts mediate sequential substrate dehydrogenations and condensations. By leveraging well‐established small‐molecule dehydrogenation chemistry into a repetitive polymer‐forming sequence, ADP unites synthetic efficiency with sustainability to offer access to structurally diverse and high‐performance polymers directly from readily available feedstocks. This minireview highlights the mechanistic foundations, representative catalytic systems, and emerging polymer architectures realized through ADP while identifying some critical challenges and future opportunities we envision will shape its evolution into an enabling platform for green and circular macromolecular design.

## Graphical abstract

Acceptor‐less dehydrogenative polymerization (ADP) enables the atom‐economic step‐growth synthesis of different type of polymers directly from simple alcohols, releasing H_2_ as the sole by‐product. This review outlines the mechanistic foundations, catalytic systems, and emerging polymer architectures of ADP, highlighting its potential as a sustainable platform for green macromolecular design.

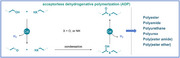

## 1 Introduction

The pursuit of polymerization methodologies to produce sustainable plastics continues to represent one of the most critical frontiers at the nexus of catalysis, macromolecular design, and materials science.^[^ ^1^, ^2^, ^3^, ^4^, ^5^ ^]^ Among established approaches, step‐growth polymerizations occupy a privileged position due to their versatility in transforming abundant building blocks, such as diols, diamines, and dicarboxylic acids into structurally diverse polymers.^[^ ^6^, ^7^, ^8^ ^]^ Yet, this strategy is historically constrained by its dependence on precise stoichiometric complementarity between reactive end groups. Even small deviations in monomer ratios can lead to sharp declines in achievable molecular weight and, by extension, undermine the material properties of the resulting polymers.^[^ ^9^ ^]^ Although such limitations may be circumvented through the design of difunctionalized monomers, varying the ratio of monomers in a polymerization to produce different materials remains a challenge.^[^ ^10^ ^]^ Thus, there remains a need for polymerization platforms capable of relaxing the rigid constraints of end‐group stoichiometry while maintaining tolerance toward variations in feed composition.

**Scheme 1:**
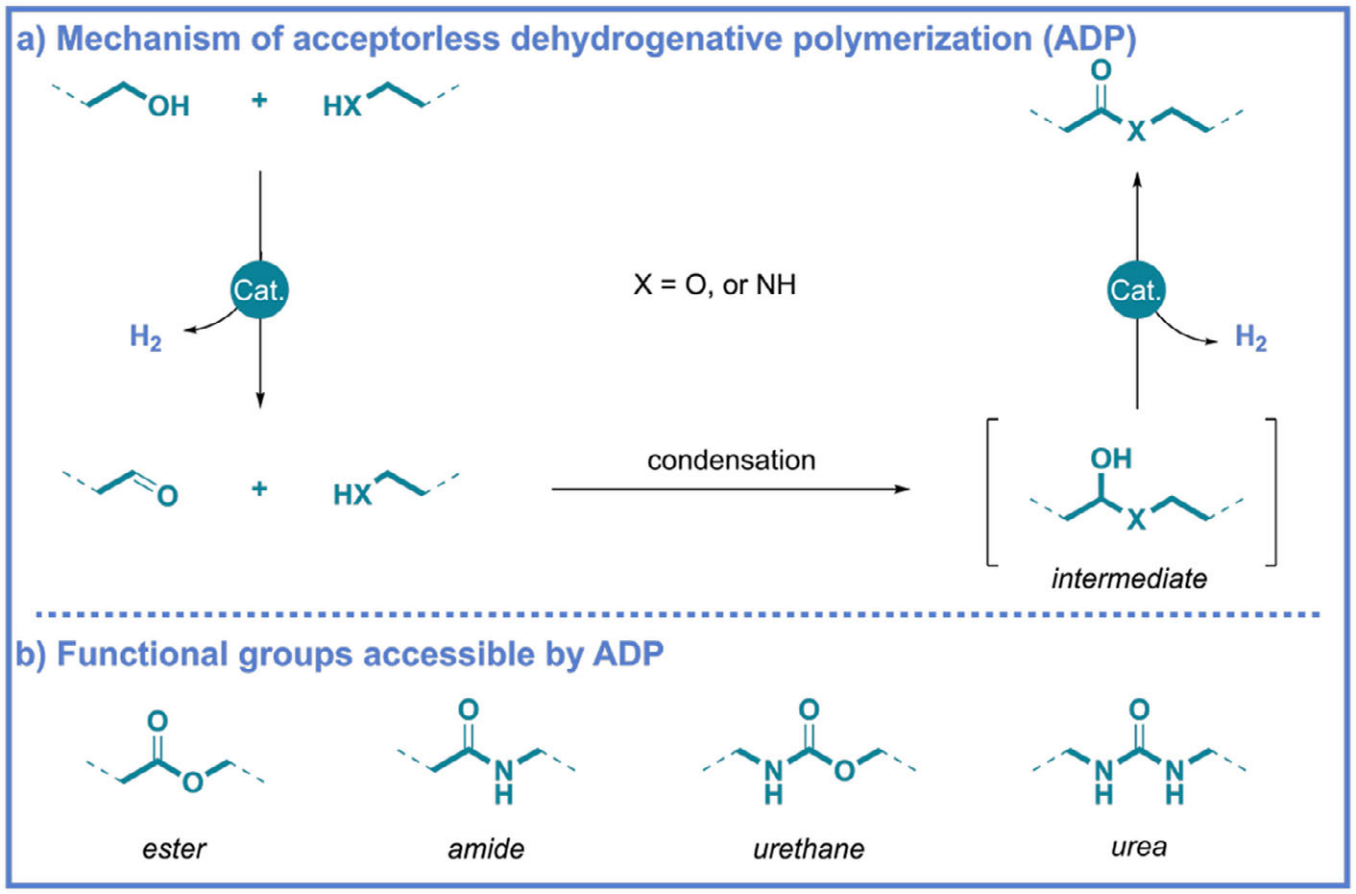
a) Mechanism of acceptorless dehydrogenative polymerization (ADP). b) Functional groups that can be synthesized via ADP.

**Scheme 2:**
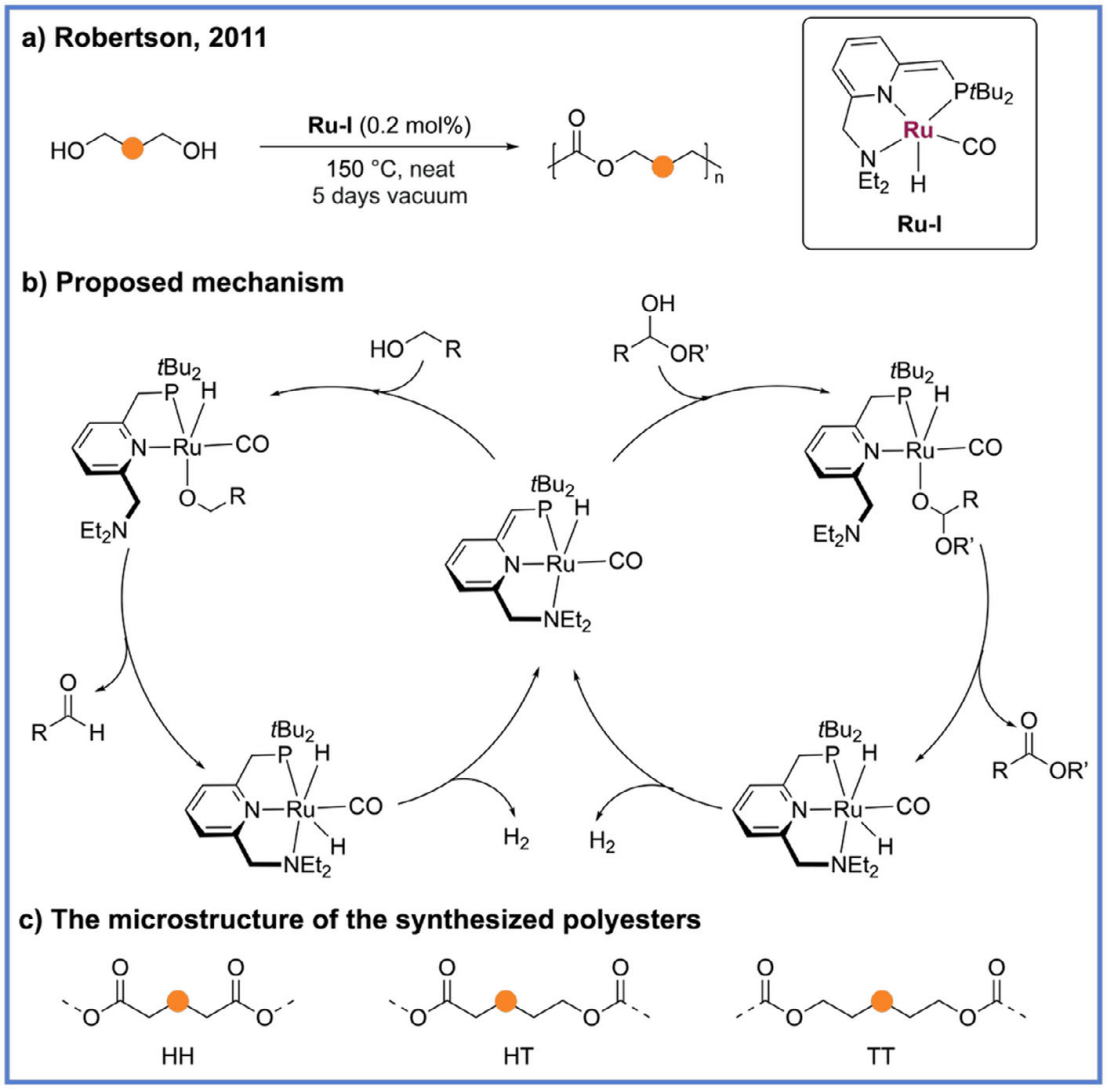
General reaction example for ADP of diols by Robertson and coworkers.

Toward addressing these considerations, acceptor‐less dehydrogenative polymerization (ADP) has emerged as an approach for polymer synthesis. By harnessing the atom‐economical coupling of unactivated alcohols and amines, ADP enables the direct construction of high‐performance polymers with molecular hydrogen gas as the only by‐product. Mechanistically, ADP is the macromolecular extension of the classical acceptor‐less dehydrogenative coupling (ADC), a powerful small molecule transformation.^[^ ^11^, ^12^, ^13^, ^14^, ^15^, ^16^ ^]^ In both processes, the transition‐metal catalyst orchestrates sequential substrate dehydrogenation and H_2_ evolution, obviating external redox reagents while achieving high atom economy and favorable thermodynamics under open systems. Whereas, ADC furnishes small‐molecule products, such as esters, amides, imines, or urea,^[^ ^17^, ^18^, ^19^, ^20^, ^21^ ^]^ ADP translates the same catalytic processes into a repetitive sequence that forges polymeric chains with controlled connectivity and architecture. Typically, the catalytic cycle proceeds through three elementary stages (Scheme 1a): (i) initial dehydrogenation, wherein coordination of an alcohol to the metal center yields a metal–alkoxide species that undergoes β‐hydride elimination to generate a carbonyl compound and a metal hydride; (ii) condensation, in which the carbonyl compound reacts with a complementary nucleophilic partner (e.g., amine, alcohol, or thiol) to form a hemiaminal or hemiacetal intermediate; and (iii) final dehydrogenation, where the intermediate undergoes a second dehydrogenation to produce the target linkage (e.g., ester or amide) and regenerate the active catalyst. Notably, the detailed dehydrogenation pathway is highly sensitive to the nature of the metal center and its supporting ligand framework. Depending on the catalyst design, the dehydrogenation step may proceed via, either an outer‐sphere, or inner‐sphere mechanism.^[^ ^22^, ^23^, ^24^ ^]^ Likewise, the second dehydrogenation of the hemiaminal or hemiacetal intermediate can follow distinct mechanistic manifolds, operating through either a concerted pathway or a stepwise pathway.^[^ ^25^, ^26^ ^]^ (scheme 2)

Through this catalytic pathway, a range of polymer classes, including polyesters, polyamides, polyurethanes, and polyureas, have been produced directly from simple and renewable feedstocks (Scheme 1b). The translation of dehydrogenative coupling principles from molecular to polymeric systems highlights not only the mechanistic robustness of ADP catalysis but also its potential to influence synthetic strategies for sustainable macromolecular design. In recent years, significant advances have been made in metal‐catalyzed platforms for dehydrogenative polymerization, and the corresponding catalysts have been summarized and discussed in recent reviews.^[^ ^27^ ^]^ This minireview will introduce the conceptual foundations and mechanistic considerations of ADP, before surveying representative catalytic systems and polymeric architectures accessible through this approach, and assess the current performance landscape of the resulting materials. Finally, we will highlight challenges and outline exciting opportunities that this emerging field may pursue in the years to come.

## 2 Polymers Synthesized via ADP and their Properties

### 2.1 Polyesters

In small‐molecule synthesis, the catalytic dehydrogenative coupling of alcohols to esters has been established as a prototypical atom‐economic and sustainable transformation, with transition metal complexes demonstrating exceptional efficiency under mild conditions.^[^ ^28^, ^29^, ^30^ ^]^ The success of primary alcohol coupling provided a conceptual basis for extending this chemistry to diols as monomers for polymer formation. In 2011, Robertson and coworkers reported the first example of ADP in which diols were polymerized in the presence of the Milstein Ru‐pincer catalyst **Ru‐I** to afford polyesters (Scheme 2a).^[^ ^31^ ^]^ In this process, high‐molecular‐weight polyesters with number‐average (*M*_n_) molecular weights up to 138,000 g mol^−1^, were obtained with low catalyst loadings (0.3 mol%) and 5 days of vacuum to remove molecular hydrogen from the reaction mixture. Evaluation of the diol substrates revealed that efficient polymerization occurred only when six or more methylene units separated the terminal hydroxyl groups, whereas shorter derivatives such as 1,5‐pentanediol preferentially underwent intramolecular cyclization to yield small molecule lactones. Mechanistically, the catalyst first mediates alcohol dehydrogenation to yield an aldehyde, which then condenses with a second alcohol molecule to generate a hemiacetal intermediate that can then undergo a second dehydrogenation to afford the ester product (Scheme 2b). The removal of H_2_ under reduced pressure drives the equilibrium toward polymer formation, therefore enabling sustained chain growth without external reagents, such as hydrogen acceptors. Furthermore, it was revealed that monomer structure exerts a decisive influence on polymer microstructure and thermal behavior. When 1,8‐octanediol is used as a monomer, each monomer could be incorporated through head‐to‐head (HH), head‐to‐tail (HT), or tail‐to‐tail (TT) linkages (Scheme 2c), resulting in spacer lengths of six, seven, or eight carbons between ester groups within the same macromolecule. This regiochemical diversity imparts a degree of region irregularity to the resulting polymer backbone, as evidenced by the depressed melting temperature (*T*_m_) observed across this class of polyesters. Such findings reveal that while the ADP process enables highly efficient and atom‐economic polycondensation, the subtle interplay of monomer symmetry and insertion mode can profoundly affect polymer crystallinity, packing, and overall thermal performance.

**Scheme 3:**
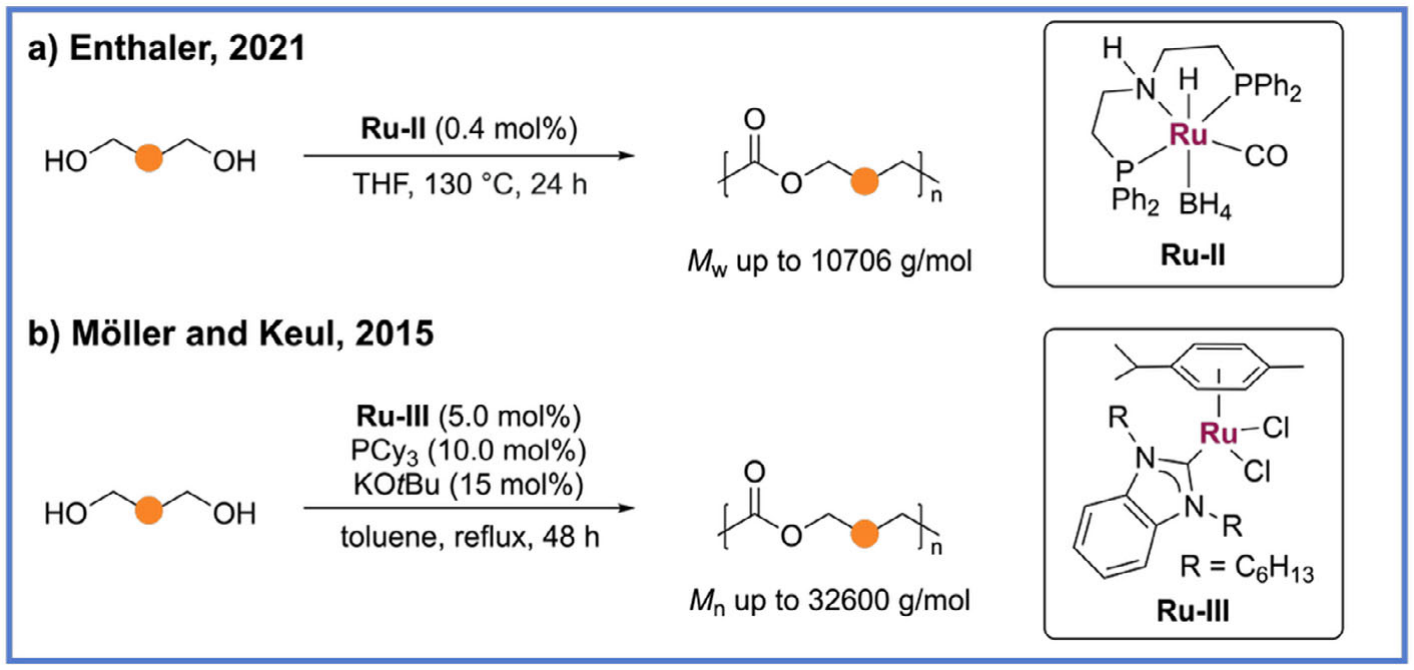
General reaction example for synthesis of polyester via ADP by using different ruthenium catalysts.

**Scheme 4:**
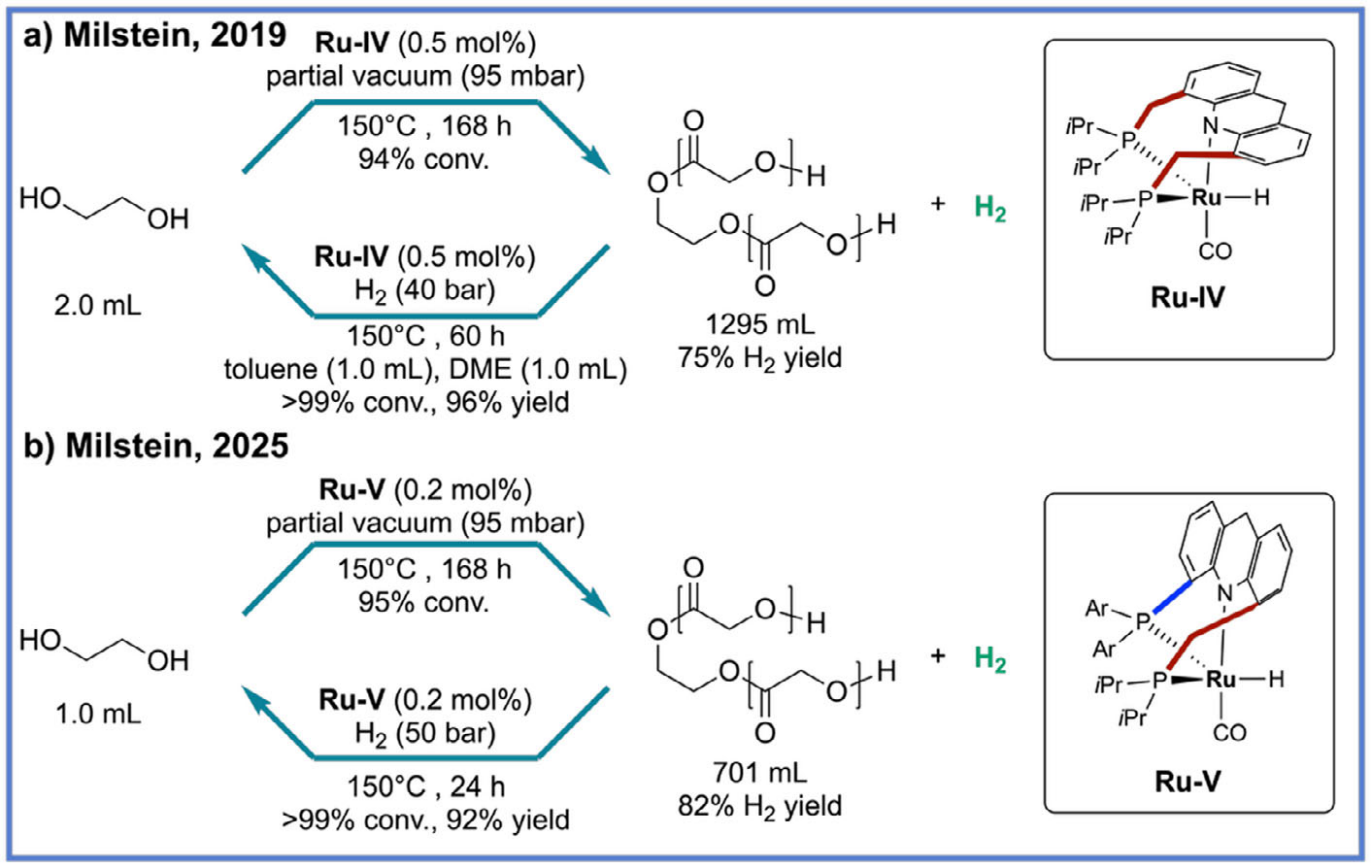
Reversible liquid‐organic hydrogen carrier developed through ADP by Milstein and coworkers.

In addition to the commercially available Milstein pincer catalyst, the well‐established Ru‐MACHO‐BH pincer complex (**Ru‐II)** has also been demonstrated as an efficient catalyst for the ADP of diols (Scheme 3a).^[^ ^32^ ^]^ When 1,6‐hexanediol was employed as the monomer, polyesters with weight‐average molecular weights (*M*_w_) up to 10 706 g mol^−1^ were obtained using 0.4 mol% **Ru‐II** at 130 °C in sealed vials under a nitrogen atmosphere after 24 h. Furthermore, this catalytic system demonstrated depolymerization of poly(ε‐caprolactone) into diols, which could subsequently be repolymerized, thereby realizing a closed‐loop recycling process for poly(ε‐caprolactone). Beyond pincer‐type metal complexes, other classes of metal complexes operate effectively in ADP. In 2015, Möller, Keul, and coworkers employed benzimidazole‐based *N*‐heterocyclic carbene (NHC) ruthenium complexes (**Ru‐III**) as alternatives to the Ru‐pincer catalyst originally reported for ADP of diols into polyesters (Scheme 4b).^[^ ^33^ ^]^ These studies revealed that conventional benzimidazole‐based NHC Ru complexes were generally inadequate for producing high‐molecular‐weight polyesters, a limitation attributed to reduced catalytic efficiency due to insufficient electron density at the metal center. To address this challenge, they introduced the electron‐rich phosphine ligand tricyclohexylphosphine (PCy_3_) into the reaction medium, employing a ligand‐exchange strategy to increase the electron density at the ruthenium center, thereby enhancing catalytic activity and enabling the synthesis of higher‐molecular‐weight polymers. In this catalytic system, the ADP of 1,12‐dodecanediol under reflux in toluene for 48 h, using 5.0 mol% **Ru‐III**, 10.0 mol% PCy_3_, and 15 mol% KO*t*Bu, yielded polyesters with an *M*_n_ of 12 100 g mol^−1^. Notably, utilizing hydroxyl‐telechelic polytetrahydrofuran (*M*_n _= ∼1000) as a monomer can produce the corresponding polyesters with *M*_n_ up to 32 600 g mol^−1^.

As catalytic platforms for the ADP of diols to polyesters have become increasingly studied, recent research has progressed toward exploiting the broader synthetic and energy‐related potential of this chemistry, for example, in hydrogen storage materials. In 2019, Milstein and coworkers introduced a new paradigm for liquid organic hydrogen carriers (LOHCs)^[^ ^34^, ^35^, ^36^ ^]^ based on a reversible polymerization‐depolymerization system that utilizes ethylene glycol (EG) as a polymer precursor (Scheme 4a).^[^ ^37^ ^]^ By employing a dearomatized acridine‐based Ru–PNP pincer complex (**Ru‐IV**), they achieved a fully reversible polymerization–depolymerization cycle that enables the interconversion of EG and its oligoester products with a remarkable theoretical hydrogen storage capacity of 6.5 wt%, exceeding both DOE and EU benchmarks. Mechanistically, the Ru complex orchestrates a series of metal–ligand cooperative transformations, initial dehydrogenation of alcohols to aldehydes, condensation to form hemiacetals, and a subsequent dehydrogenation yielding ester linkages while liberating H_2_. This process proceeds efficiently under base‐ and solvent‐free conditions, achieving up to 94% conversion and 99.6% hydrogen purity, while density functional theory (DFT) analyses reveal a nearly thermoneutral energy profile that rationalizes the system's exceptional reversibility, atom economy, and energy efficiency. Notably, in 2025, the same group advanced the system by developing long–short‐arm acridine Ru–PNP pincer catalysts (**Ru‐V**), which combine structural rigidity and flexibility to overcome the dual challenge of catalyst stability and late‐stage dehydrogenation efficiency (Scheme 4b).^[^ ^38^ ^]^ The optimized complex **Ru‐V** achieved > 99% conversion and up to 95% H_2_ yield under the same conditions, corresponding to a hydrogen storage capacity of 6.2 wt%. Mechanistic and DFT studies uncovered that the unique mer‐fac fluxionality of the long–short‐arm architecture reduces the activation barrier for β‐hydride elimination (Δ*G* ^‡^ ≈ 20 kcal mol^−1^) compared to earlier Ru systems, enabling more efficient conversion of 2‐hydroxyethyl glycolate to oligomers. Importantly, the hydrogenation proceeds efficiently under solvent‐free conditions, delivering even higher yields (96%) and thereby emphasizing the system's reversibility and strong industrial relevance.

**Scheme 5:**
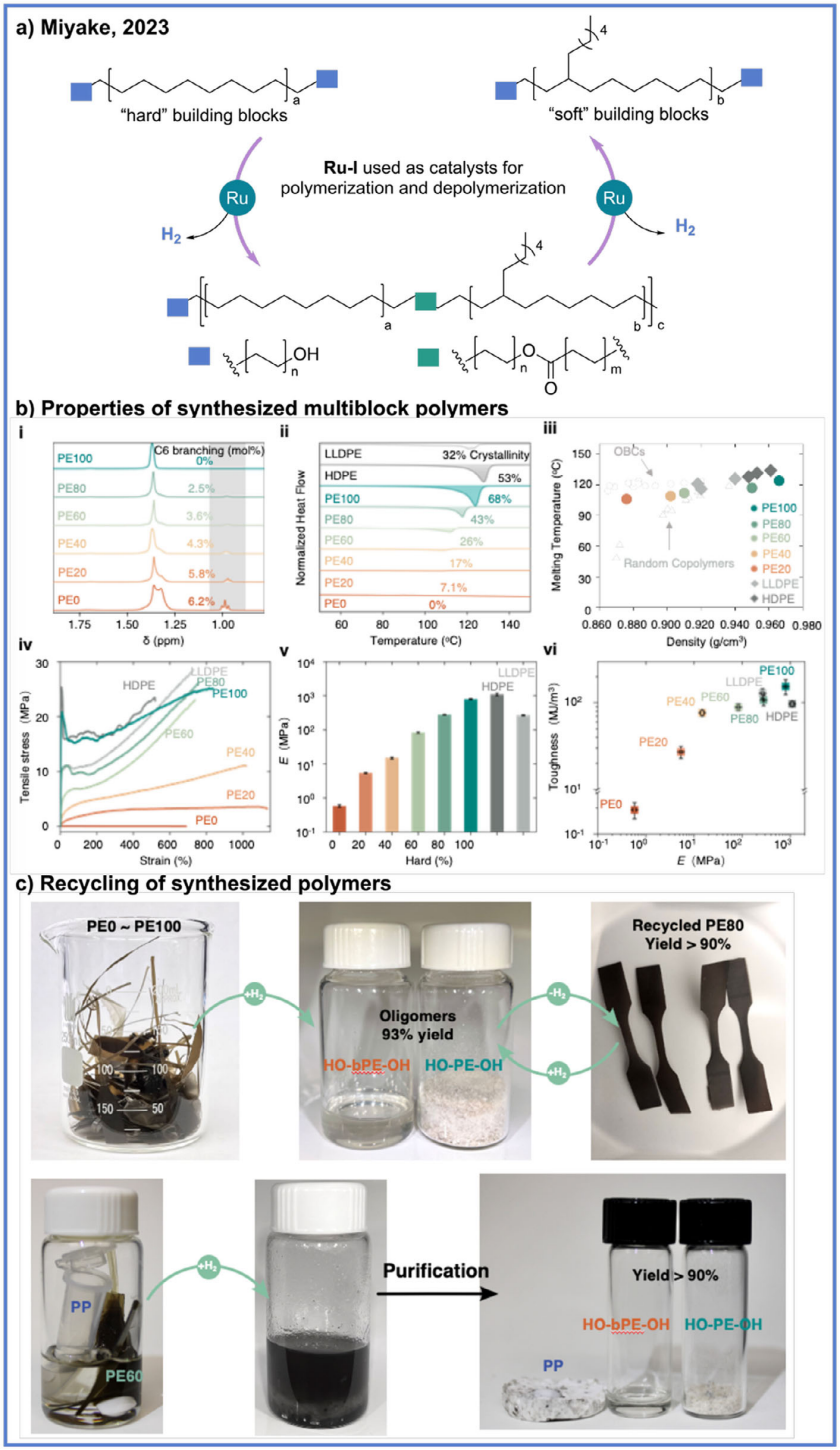
Recycling polyolefin‐like materials synthesized through ADP by Miyake and coworkers. Copyright 2023, The American Association for the Advancement of Science.

Beyond impressive success in LOHCs, the ADP strategy has also achieved significant advances in controlling polymer structure and enabling chemical reversibility. In 2023, Miyake and coworkers reported the synthesis of chemically recyclable polyolefin‐like multiblock polymers via ADP, achieving materials that targeted the mechanical robustness and diversity of commodity polyolefins with chemical recyclability (Scheme 5a).^[^ ^39^ ^]^ By coupling telechelic diol oligomers of crystallizable “hard” and amorphous “soft” segments through a ruthenium pincer complex (**Ru‐I**), high–molecular‐weight multiblock polymers (*M*_w_ = 62–90 kDa) were obtained with tunable block compositions and corresponding property profiles. The resulting materials exhibited high melting transitions (up to 124 °C) and low glass‐transition temperatures (down to –60 °C), endowing them with a unique combination of strength, elasticity, and processability across a broad temperature window comparable to and even exceeding that of commercialized olefin block copolymers (OBCs) (Scheme 5b, iii). Furthermore, the mechanical performance of these multiblock polymers is particularly noteworthy, with Young's modulus and tensile strength spanning over three orders of magnitude, as the hard‐block fraction increases, while elongation at break consistently exceeds 700% and even reaches over 1000% for intermediate compositions. These materials also demonstrate tensile toughness values up to 150 MJ m^−3^, rivaling or surpassing high‐performance engineering plastics while retaining full thermoplastic reprocessability (Scheme 5b, iv–vi). A key feature of this system is the potential for chemical recyclability of these different multiblock polymers, even in the presence of other commodity plastics (Scheme 5c). Under 40 bar H_2_ and in the presence of the same Ru catalyst, these multiblock polymers of different hard and soft compositions could be combined, and the ester linkages underwent hydrogenolysis to regenerate the original diol building blocks in > 99% yield within 24 h. The recovered monomers were repolymerized under identical conditions to afford polymers with nearly identical molecular weights, dispersities, and mechanical properties over at least three cycles. The depolymerization was selective even in mixed‐plastic environments containing isotactic polypropylene (*i*PP) where the ADP‐derived multiblock polymers were quantitatively recovered while *i*PP remained intact.

**Scheme 6:**
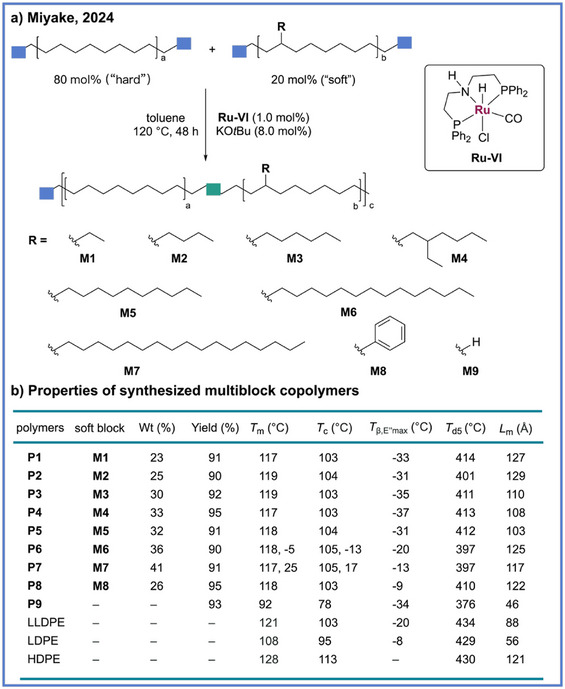
Synthesis of polyethylene‐like multiblock copolymers with tunable branched structures via ADP from Miyake and coworkers.

Building on this multiblock polymer approach through ADP, Miyake and coworkers further leveraged this platform by introducing control over the branch architecture within the soft segments, enabling systematic modulation of the thermal, mechanical, and interfacial properties of polyethylene‐like multiblock copolymers.^[^ ^40^ ^]^ In this design, a series of branched soft‐block oligomers were synthesized via chain‐transfer ring‐opening metathesis polymerization followed by hydrogenation and subsequently coupled with crystallizable hard blocks through Ru‐catalyzed (**Ru‐IV** used as catalyst) ADP (Scheme 6a). The resulting polymers could exhibit high melting transition temperatures (117 °C–119 °C) and tunable glass‐transition temperatures (−37 °C– − 9 °C), with the variation in branch topology dictating the balance between chain mobility and segmental packing (Scheme 6b). Such architectural diversity allowed predictable tuning of elasticity, modulus, and toughness while maintaining the orthorhombic crystalline phase characteristic of polyethylene, thereby preserving the desirable mechanical integrity of conventional polyolefins. Notably, these multiblock copolymers achieved mechanical performances on par with or surpassing those of commercial LLDPE and LDPE, featuring Young's moduli of 240–370 MPa, elongations at break exceeding 400%–600%, and toughness values up to 70 MJ m^−3^.

**Scheme 7:**
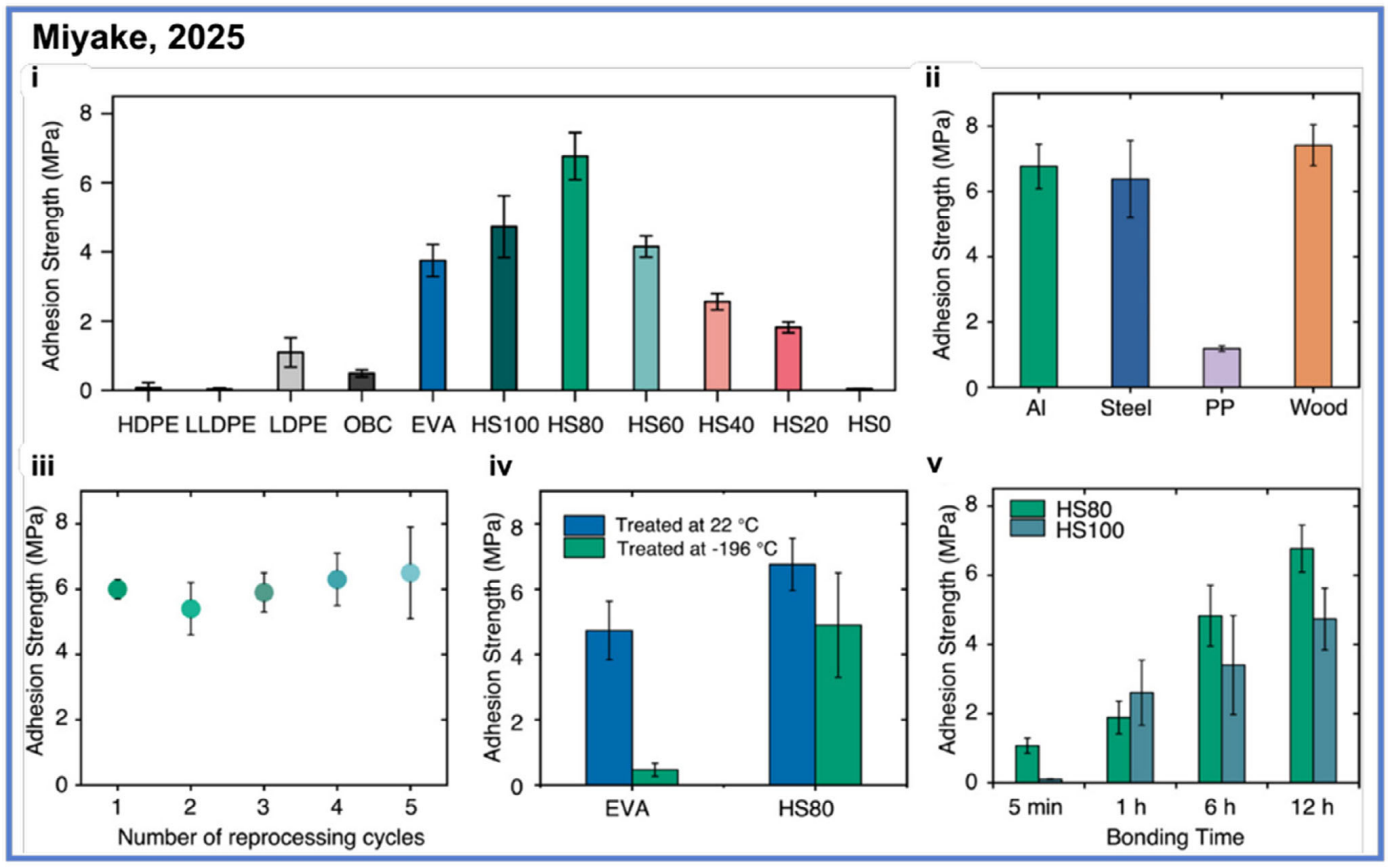
Chemically recyclable multiblock copolymers as hot‐melt adhesive from Miyake and coworkers. Copyright © 2025 John Wiely & Sons, Inc.

Intriguingly, despite incorporating only trace amounts of ester functionality (<1%), these multiblock polymers exhibited remarkably enhanced adhesion to metal substrates, highlighting how subtle chemical modifications can profoundly influence interfacial interactions relative to nonpolar polyolefins.^[^ ^40^ ^]^ Building upon this insight, Miyake and coworkers advanced their ADP‐derived multiblock polyolefin framework toward the design of chemically recyclable hot‐melt adhesives that simultaneously deliver polyolefin‐like mechanical robustness, while the ester functionality imparts tunable interfacial adhesion and closed‐loop recyclability.^[^ ^41^ ^]^ Through architectural control over the crystalline “hard” and amorphous “soft” segments containing < 1% ester linkages, they created materials exhibiting tunable viscoelastic properties and exceptional shear strengths up to 6.8 MPa on aluminum, exceeding those of commercial EVA and OBC benchmarks (Scheme 7). Importantly, this modular approach revealed a quantitative structure–property–function relationship in which systematic variation of soft‐block content and branching density enabled predictive optimization of cohesive energy density, surface wetting, and interfacial adhesion, thereby bridging molecular design with macroscopic performance. Notably, both the adhesive layer and the laminated substrates (e.g., *i*PP/Nylon composites) could be selectively depolymerized under mild hydrogenation conditions and fully reconstructed without any loss of strength or toughness.

**Scheme 8:**
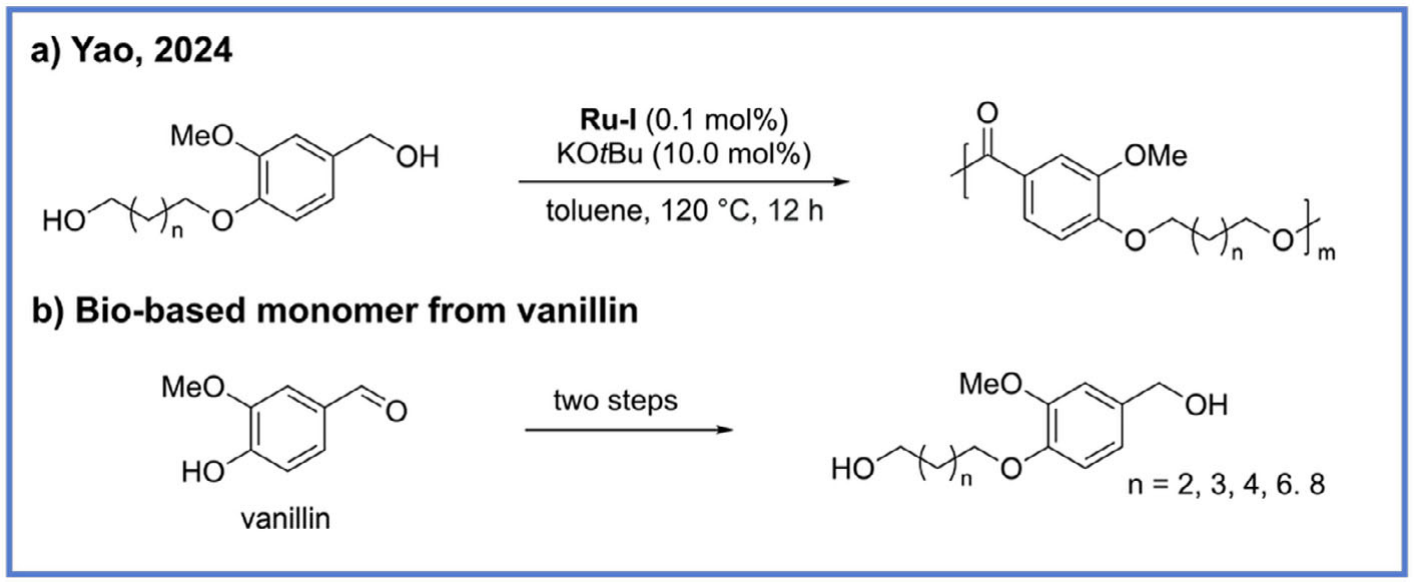
Bio‐based alipharomatic polyesters using vanillin‐derived bifunctional hydroxy‐aldehyde monomers from Yao and coworkers.

As diols represent one of the most accessible and versatile biomass‐derived building blocks motivating use for sustainable polymer synthesis, recent advances in ADP have increasingly leveraged renewable feedstocks to construct high‐performance and recyclable materials. In 2024, Yao and coworkers reported an approach to synthesize biobased, chemically recyclable polyesters from vanillin‐derived hydroxy‐aldehyde monomers via ADP (Scheme 8).^[^ ^42^ ^]^ In this work, **Ru‐I** was used as the catalyst to synthesize vanillin‐based polyesters with high‐molecular‐weights (*M*_n_ up to 31.0 kDa), excellent thermal stability (*T*_d5_ > 350 °C), and tunable thermal transitions dictated by the aromatic or aliphatic character of the monomers. Notably, the materials exhibited closed‐loop chemical recyclability as selective hydrogenative depolymerization quantitatively regenerated the original monomers.

**Scheme 9:**
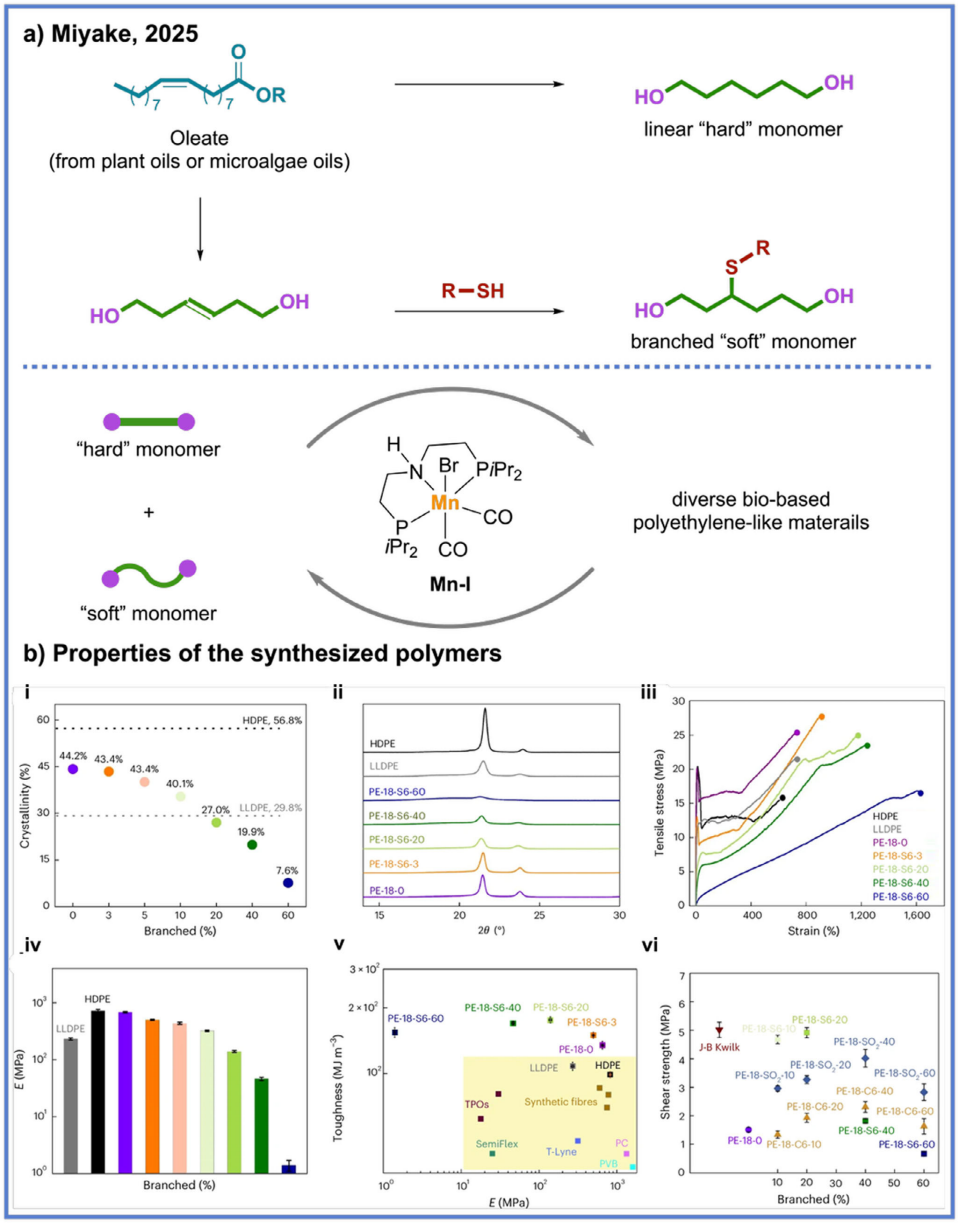
Synthesis of bio‐based polyethylene‐like material via ADP from Miyake and coworkers. Copyright © 2025 Spring Nature.

In 2025, Miyake and coworkers built upon their previous work on ruthenium‐catalyzed ADP for recyclable polyolefin‐like materials,^[^ ^34^, ^35^, ^36^ ^]^ as well as work on bio‐based diol synthesis and polymerization from the Mecking group^[^ ^43^ ^]^ to report a manganese‐catalyzed, closed‐loop platform for producing polyethylene‐like polymers with tunable performance (Scheme 9).^[^ ^44^ ^]^ In this study, both linear and branched aliphatic diols, which are readily obtainable from biomass (Scheme 9a top), serve as feedstocks for the ADP catalyzed by an earth‐abundant Mn complex (**Mn‐I**), thereby eliminating the need for precious metals while maintaining high catalytic efficiency. Variations in the feed ratios of the two diols result in materials with exceptional tunability in thermal and mechanical properties, leading to versatile properties that span the full range of thermoplastic to plastomer to elastomer characteristics (Scheme 9b). Polymers derived from linear diols display high crystallinity, with *T*_m_ up to 94 °C, and mechanical strength and modulus properties that resemble those of high‐density polyethylene (HDPE). In contrast, polymers synthesized from branched diols show reduced crystallinity but dramatically enhanced toughness and elasticity, achieving elongation at break exceeding 1600% and tensile toughness values up to 180 MJ m^−3^, while maintaining outstanding thermal stability (*T*_d5_ > 360 °C). Moreover, the incorporation of controlled branching effectively modulates the viscoelastic response of the materials and significantly improves adhesion to metallic and polar substrates, resulting in extended potential applications beyond structural plastics to functional coatings, pressure‐sensitive adhesives, and flexible packaging.

**Scheme 10:**
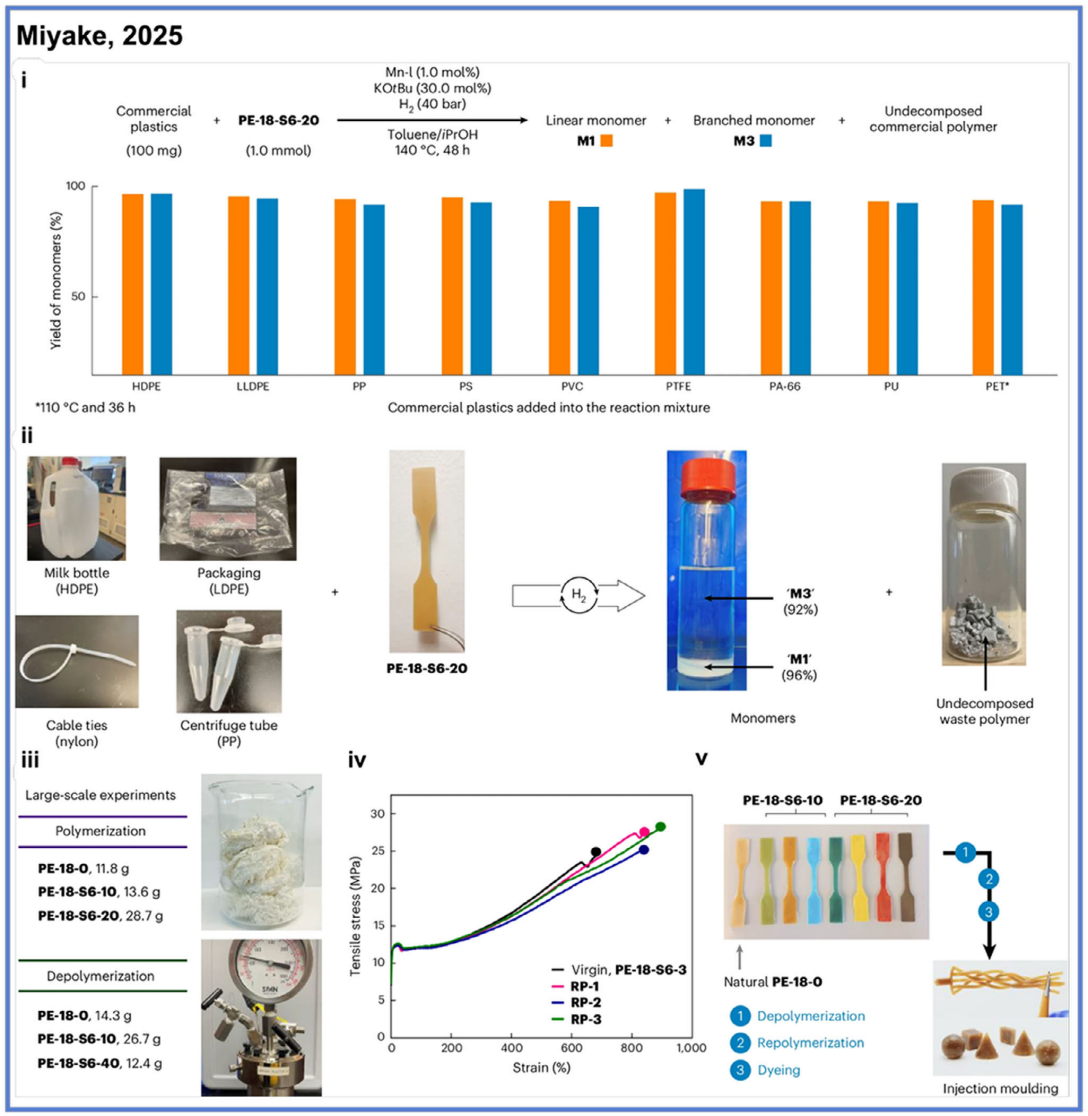
Catalytic recycling of bio‐based polyethylene‐like materials. Copyright © 2025 Spring Nature.

Beyond their tunability in mechanical and thermal properties, these polymers also exhibit closed‐loop chemical recyclability (Scheme 10). Using the same manganese complex that mediates polymer formation, Miyake and coworkers also demonstrated that the synthesized materials could be depolymerized back to monomer under mild conditions with monomer recovery yields as high as 95%, even in the presence of commercial plastics (Scheme 10, i). This depolymerization proceeded cleanly and with high selectivity while maintaining full efficiency despite the presence of colorants, plasticizers, stabilizers, and mixed polymer streams (Scheme 10, ii). Moreover, repeated polymerization–depolymerization cycles yielded materials with maintained mechanical performance (Scheme 10, iv) compared to the original material, therefore supporting the robust reversibility of the process. These features, combined with the system's scalability and compatibility with injection molding, enable the fabrication of bulk polymeric components that retain both structural integrity and recyclability (Scheme 10, v).

**Scheme 11:**
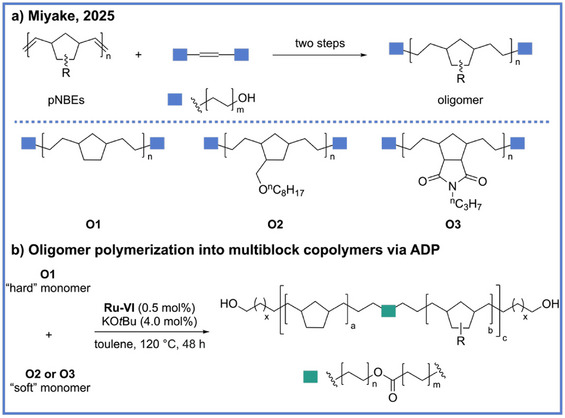
Upcycling polynorbornene derivatives into multiblock materials from Miyake and coworkers.

In addition to utility in synthesizing recyclable polymers, the ADP strategy has recently been extended to the upcycling of existing polymeric materials. In 2025, Miyake and coworkers reported an approach for transforming polynorbornene (pNBE) derivatives into chemically recyclable multiblock linear and thermoset plastics via a tandem olefin metathesis–ADP sequence (Scheme 11).^[^ ^45^ ^]^ This strategy provides a powerful blueprint for converting traditionally difficult‐to‐recyclable polymers into more easily recycled materials with tailored architectures and tunable thermomechanical properties. In this study, pNBE derivatives were first deconstructed through olefin metathesis to yield oligomeric fragments containing repeating structural motifs and alcohol chain‐end groups (Scheme 11a). These fragments were subsequently reassembled through ADP into linear or cross‐linked multiblock polymers, effectively preserving or even enhancing the desired combination of strength, elasticity, and thermal stability (Scheme 11b). The upcycled materials exhibited diverse and controllable mechanical profiles, featuring high melting transition temperatures and broad glass‐transition temperature ranges, which could be precisely modulated by adjusting the feed ratio of the oligomeric building blocks. Moreover, consistent with the group's earlier work on recyclable polyolefin‐like materials,^[^ ^39^, ^44^ ^]^ these materials could be depolymerized back to monomers under mild conditions and repolymerized without degradation of properties.

**Scheme 12:**
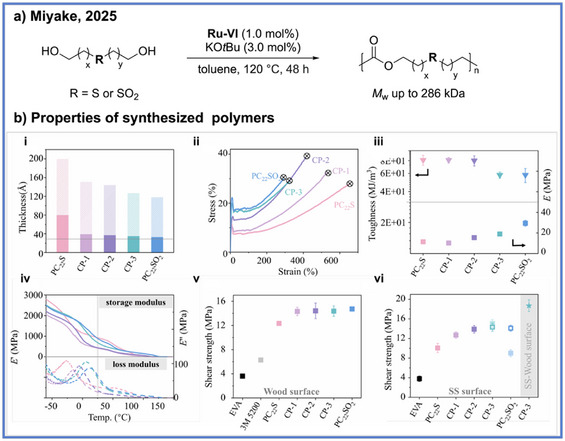
Synthesis of sulfur‐based polyester via ADP from Miyake and coworkers. Copyright © 2025 John Wiely & Sons, Inc.

Most recently, Miyake and coworkers have introduced sulfur‐containing functional groups, such as sulfone and sulfide moieties, into long‐chain diol monomers, enabling the synthesis of high‐performance hot‐melt adhesives via ADP (Scheme 12).^[^ ^46^ ^]^ The resulting materials exhibit shear strengths exceeding 14–15 MPa on both wood and stainless‐steel substrates, approaching the performance range of structural thermoset adhesives. A key insight from this study is that the spatial localization of polar groups within high‐modulus crystalline domains, rather than within amorphous regions, dramatically enhances stress transfer efficiency and thereby maximizes both cohesive and interfacial adhesion forces to allow higher performing adhesives. Beyond their mechanical performance, these sulfur‐based polymers demonstrate excellent oxygen and water‐vapor barrier properties, supporting their use in demanding applications such as microelectronic encapsulation. Importantly, the materials can undergo closed‐loop chemical recycling through mild hydrogenative depolymerization, enabling efficient monomer recovery even from complex mixed‐material systems.

### 2.2 Polyamides and Polyurethanes

**Scheme 13:**
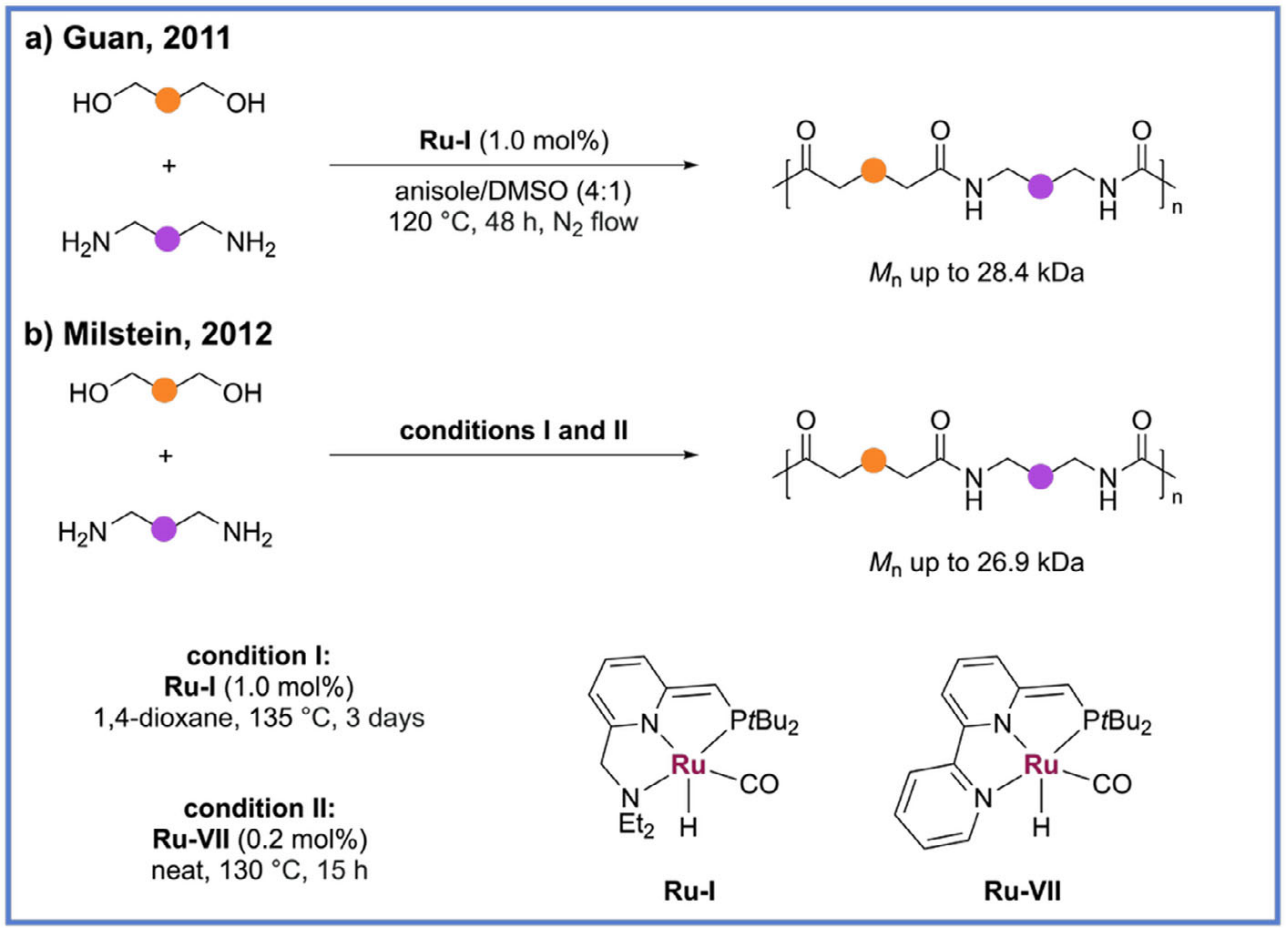
General reaction example for synthesis of polyamide via ADP.

In analogy to the ADC of alcohols to esters, the coupling of alcohols with amines can afford amides.^[^ ^47^, ^48^ ^]^ Accordingly, the application of ADP to polyamide synthesis represents a viable and conceptually elegant strategy for constructing nitrogen‐containing macromolecules. In 2011, Guan and coworkers reported the first direct catalytic synthesis of polyamides via the ADC of diols and diamines, marking a conceptual breakthrough in atom‐economical amide bond formation (Scheme 13a).^[^ ^49^ ^]^ In this work, both aliphatic and aromatic, as well as linear and cyclic diols and diamines, were successfully polymerized in the presence of 1.0 mol% Milstein Ru‐pincer catalyst (**Ru‐I**) to afford different types of polyamides (*M*_n_ up to 28,400 kDa). Owing to the high catalytic selectivity for primary amines over secondary amines, polyamines could be readily incorporated into linear polyamides without the need for laborious protection/deprotection steps. Unlike traditional polyamidation, which requires stoichiometric activation reagents (e.g., acid chlorides, coupling agents) or high‐temperature melt condensation, this catalytic route proceeds under relatively mild conditions with molecular hydrogen as the sole byproduct, achieving excellent selectivity and sustainability. Notably, due to the extensive hydrogen bonding between amide linkages, the resulting polyamides exhibited limited solubility in common solvents such as toluene, which prematurely inhibited chain growth. To overcome this challenge, a mixed solvent system of anisole and DMSO (6:1) was employed, enabling improved polymerization performance and synthesis of higher molecular weight polymers.

In a further advancement, Milstein and coworkers demonstrated that two dearomatized ruthenium complexes (**Ru‐I** and **Ru‐VII**) could efficiently catalyze the ADP of diols and diamines to synthesize polyamides (Scheme 13b).^[^ ^50^ ^]^ Their approach broadened the substrate scope to include aliphatic, aromatic, and heteroaromatic monomer combinations, achieving polyamides with *M*_n_ up to 26.9 kDa and high thermal stability (*T*_d_ up to 680 °C). Importantly, the reactions were conducted under mild conditions without additives and even under solvent‐free melt conditions using as little as 0.2 mol % catalyst, highlighting the system's scalability and industrial relevance.

Notably, the potentially competing polyester formation via acceptor‐less dehydrogenative self‐polymerization of alcohols was not observed in either study. This observation can be attributed to the fact that the intermediate aldehyde preferentially reacts with the amine (being a stronger nucleophile than the alcohol) to form a hemiaminal intermediate (rather than a hemiacetal), which subsequently undergoes dehydrogenation to afford the amide.^[^ ^51^ ^]^ Moreover, it should be considered that the Milstein Ru catalyst is also competent in catalyzing amide formation through the coupling of esters with amines.^[^ ^52^ ^]^ Thus, even if esters were to be generated, they could be further converted into polyamides under these reaction conditions.

**Scheme 14:**
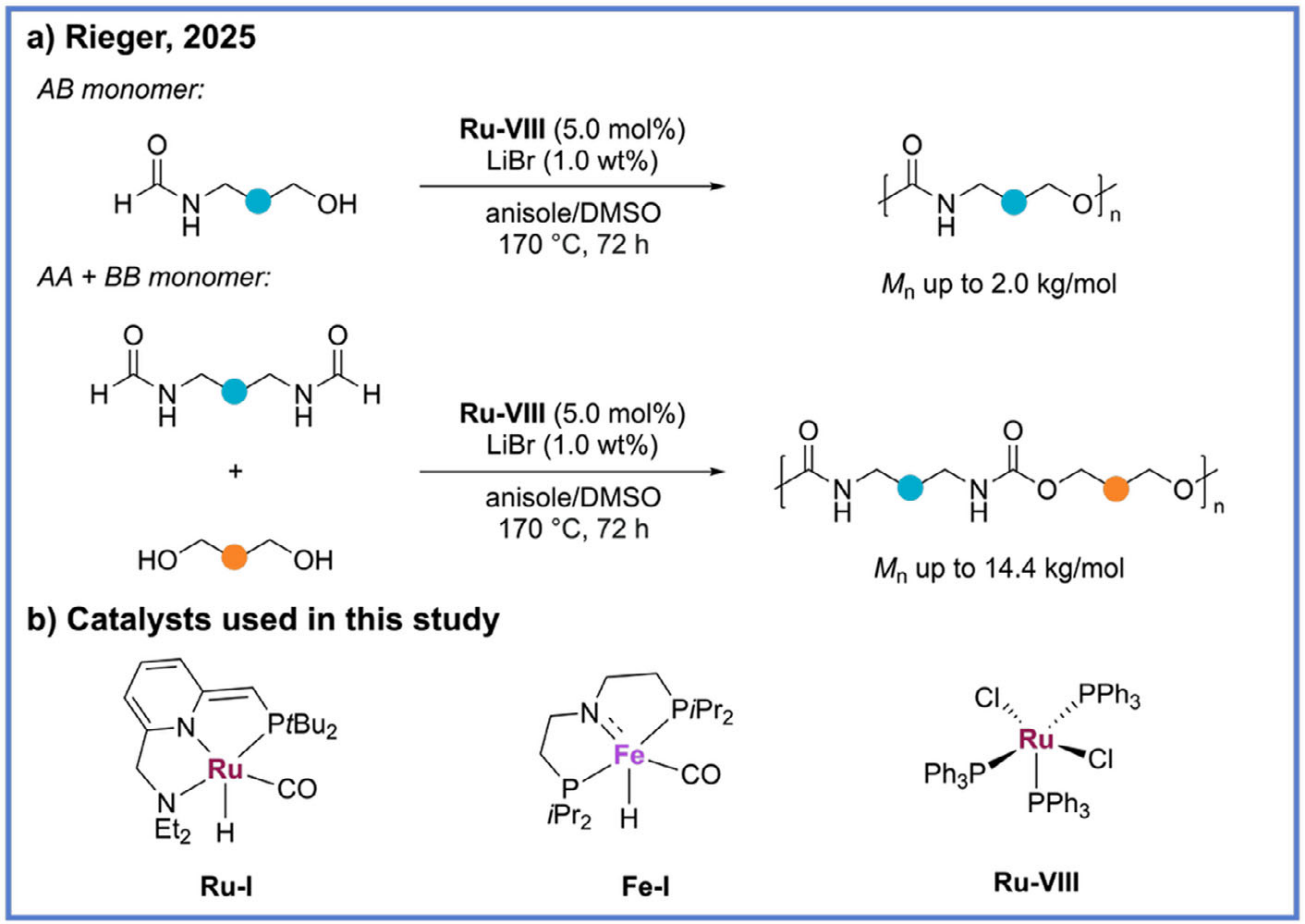
Synthesis of polyurethanes from formamides and alcohols via ADP from Rieger and coworkers.

Building on the success of polyamide synthesis via ADP, recent efforts have sought to extend this catalytic approach to the synthesis of polyurethanes, a class of polymers traditionally dependent on hazardous isocyanate or phosgene chemistry.^[^ ^53^, ^54^, ^55^ ^]^ In 2025, Rieger and coworkers reported an approach to polyurethanes through the ADP of formamides and diols, with the key monomeric building blocks themselves accessible from formic acid as a renewable carbonyl source (Scheme 14a).^[^ ^56^ ^]^ In this study, both ruthenium‐and iron‐based pincer type catalysts (**Ru‐I** and **Fe‐I**) were found competent for this transformation, yet the application of a nonpincer Ru catalyst (**Ru–VII**) afforded significantly higher molecular‐weight polyurethanes, thus challenging the notion that pincer‐type complexes are critical for efficient ADP reactions (Scheme 14b). Under optimized conditions, both AB‐type and AABB‐type monomers were polymerized to yield high‐molecular‐weight polyurethanes (*M*_n_ up to 14.4 kDa) that exhibit thermal decomposition temperatures (*T*_d5_ ≈ 320 °C) and mechanical performance on par with commercial polyurethane benchmarks. Notably, beyond its synthetic elegance, this work demonstrates the dual role of formic acid as both a carbonyl and hydrogen donor.

### 2.3 Polyureas

**Scheme 15:**
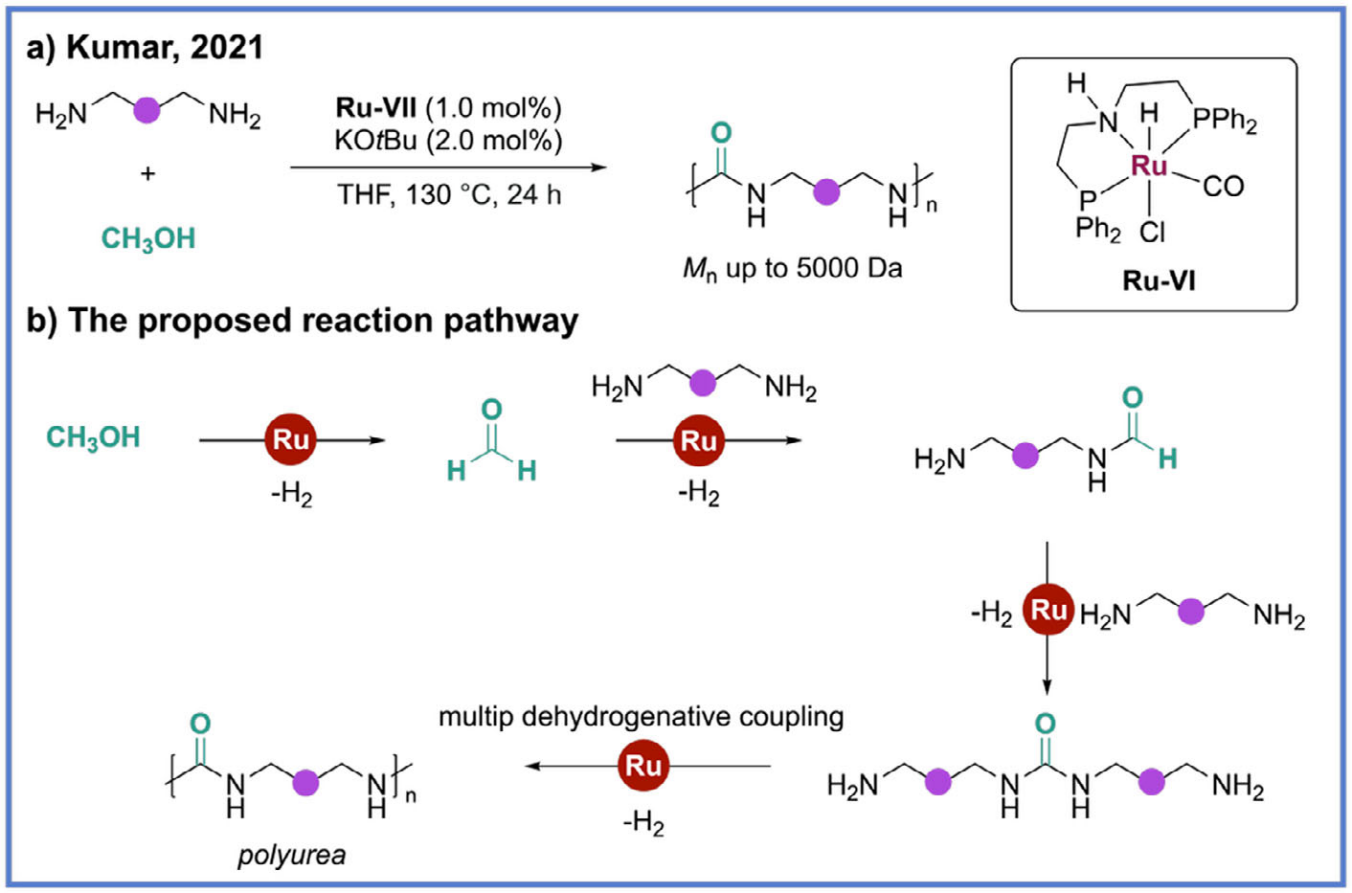
Synthesis of polyurea from diamine and methanol via ADP from Kumar and coworkers.

The ADP strategy has also been extended beyond esters, amides, and polyurethanes to the synthesis of polyureas. In 2021, Kumar and coworkers reported the first example of polyurea synthesis via ADP using Ru complexes (**Ru‐VI**) as catalysts (Scheme 15a).^[^ ^57^ ^]^ They demonstrated that diamines and methanol, two abundant feedstocks, can be directly coupled to form polyureas under mild conditions. This approach replaces the conventional reaction of diamines with toxic diisocyanates or phosgene‐derived intermediates,^[^ ^58^, ^59^ ^]^ offering a greener, atom‐economical, and phosgene‐free alternative. Mechanistically, the reaction proceeds through sequential dehydrogenation and condensation steps: methanol is first converted to formaldehyde, which reacts with amines to form hemiaminal intermediates, followed by further dehydrogenation to generate urea linkages with the liberation of H_2_ (Scheme 15b). The optimized catalytic system employing 1.0 mol% **Ru‐VI** complex and 2.0 mol% KO*t*Bu at 130 °C achieved yields up to 97% and *M*_n_ up to 5500 Da, producing thermally robust polyureas with *T*_d_ in the range of 230 °C–299 °C. Notably, the process was effective for a broad range of diamines, including aliphatic, aromatic, and even bio‐based substrates, thereby allowing for the preparation of renewable and chiral polyureas that hold potential applications in coatings, adhesives, and biomedical materials. The authors further demonstrated the first synthesis of a ^13^C‐labelled polyurea using ^13^CH_3_OH, highlighting the method's versatility for isotopic labelling, drug‐delivery tracking, and microplastic fate studies.

**Scheme 16:**
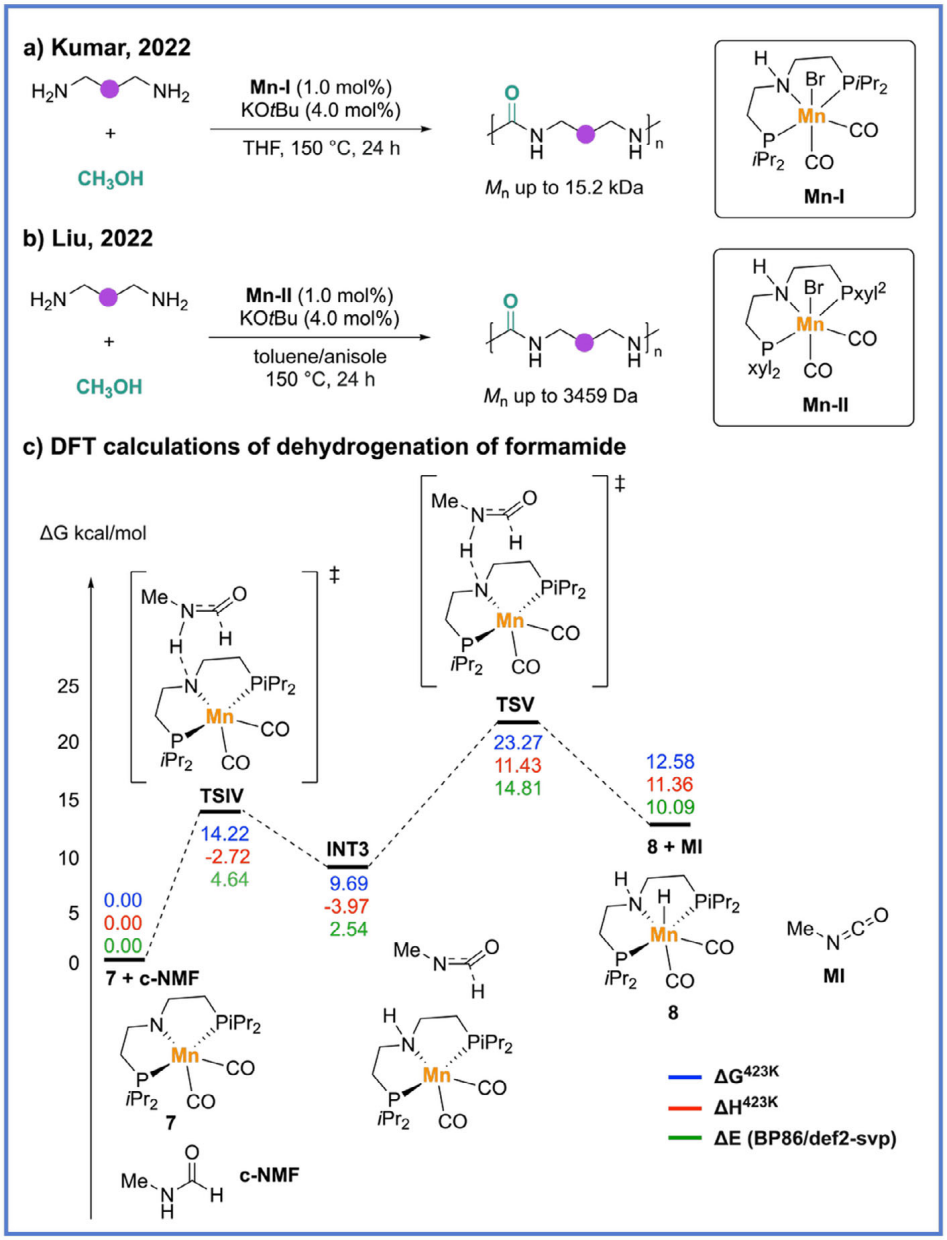
Synthesis of polyurea from diamine and methanol via ADP by using two different manganese catalytic systems.

Following the pioneering ruthenium‐based work that first established the feasibility of ADP to produce polyureas from methanol and diamines, subsequent research efforts have focused on improving the sustainability and economic viability of this chemistry by replacing noble metals with earth‐abundant alternatives. In 2022, two groups (Kumar and Bühl group,^[^ ^60^ ^]^ and Liu groups^[^ ^61^ ^]^) reported the polyurea synthesis via ADP by using an Mn pincer complex in combination with catalytic KO*t*Bu at the same time (Scheme 16). Both groups independently proposed a consistent mechanistic framework and conducted detailed investigations into the key step of urea bond formation, namely, the transformation from formamide to urea. Control experiments from both studies revealed that neither secondary amines nor *N*,*N*‐disubstituted formamides yielded urea products, while formamides bearing an N–H proton readily underwent coupling to generate urea. Furthermore, the reaction of *N*‐methylformanilide with primary amines afforded symmetric ureas along with *N*‐methylaniline as a byproduct, collectively supporting an isocyanate‐mediated pathway. For further support, Kumar and coworkers substantiated this mechanistic hypothesis through DFT calculations (Scheme 16c). Their analysis indicated that dehydrogenation of formamide proceeds via a zwitterionic intermediate and a *β*‐hydride elimination transition state that possessed an activation barrier of Δ*G* ^‡^ = 23.3 kcal mol^−1^. The regeneration of the catalytically active Mn–hydride species was identified as the rate‐determining step (Δ*G* ^‡^ = 30.6 kcal mol^−1^) in this mechanism. The subsequent reaction between the in situ–formed isocyanate and amine to furnish the urea linkage was found to be exergonic (Δ*G* = –3.5 kcal mol^−1^), therefore supporting the thermodynamic favorability of this mechanistic pathway.

The successful synthesis of polyureas from diamines and methanol highlighted the key role of formamide intermediates in dehydrogenative polymerization, inspiring further exploration of direct polyurea formation from diformamides and diamines. In 2023, Kumar, Tan, and coworkers achieved this transformation using a manganese pincer complex (**Mn‐I**) as a catalyst, enabling the ADP of diformamides and diamines to produce polyureas under mild conditions.^[^ ^62^ ^]^ The resulting materials exhibited outstanding thermal and mechanical robustness (*T*_m_ up to 221 °C and *T*_d_ up to 266 °C) while exhibiting modulus and hardness values in the gigapascal range. Notably, the polymerization demonstrated broad substrate tolerance by effectively polymerizing both aliphatic and aromatic diformamides.

**Scheme 17:**
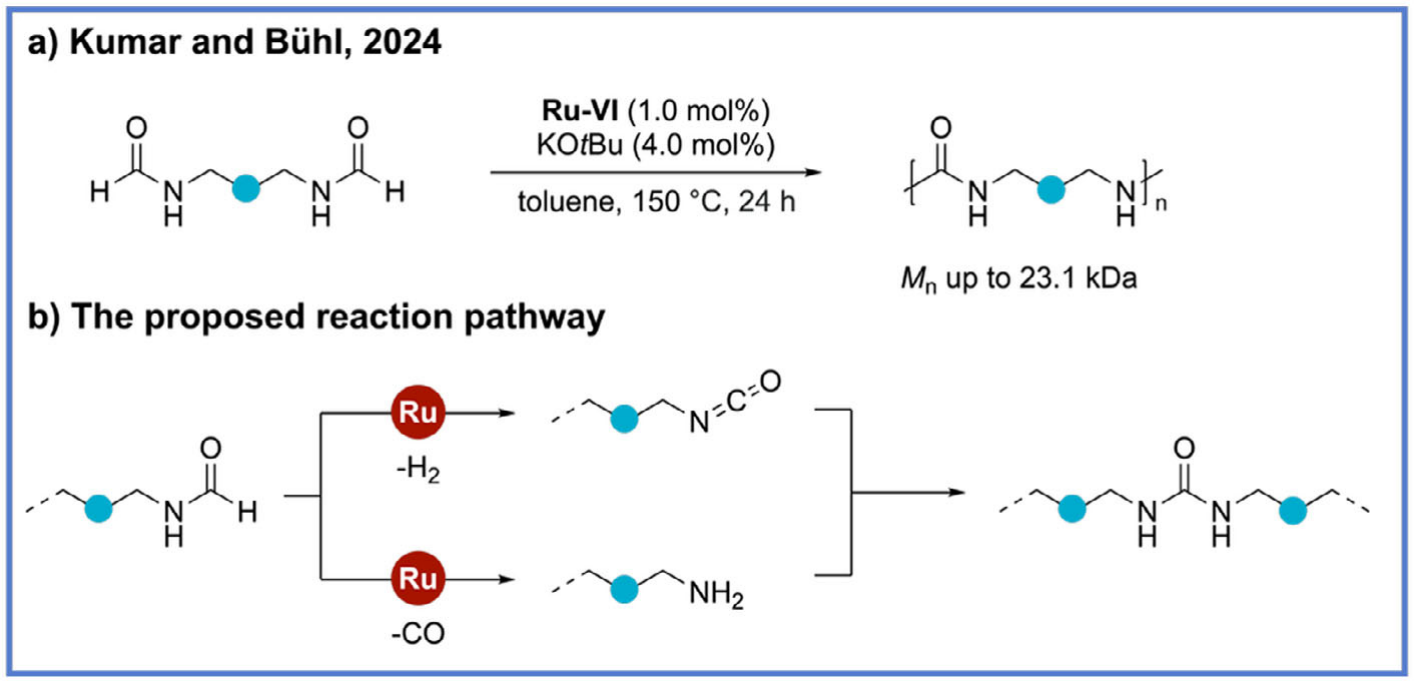
Synthesis of polyurea from diformamides via ADP and decarbonylation from Kumar, Bühl, and coworkers.

Interestingly, polyureas can also be synthesized directly from diformamide without diamine. In 2024, Kumar, Bühl, and coworkers reported a ruthenium‐catalyzed (**Ru‐VI**) dehydrogenative and decarbonylative polymerization strategy that enables the isocyanate‐ and diamine‐free synthesis of polyureas directly from diformamides (Scheme 17a).^[^ ^63^ ^]^ This methodology exploited the self‐coupling of formamides to construct polyurea backbones while releasing carbon monoxide (CO) and H_2_ as the only by‐products (Scheme 17b). The resulting polymers exhibit remarkably high molecular weights (*M*_n_ up to 23.1 kDa and exceeding the 55 kDa calibration limit in one instance) and exceptional thermal robustness (*T*_m_ reaching 222 °C and *T*_d5_ ∼298 °C) values comparable to advanced engineering plastics. Detailed mechanistic and DFT studies elucidated a complex catalytic network involving formamide N–H activation, and transient isocyanate intermediate emergence, collectively underpinning the efficiency and selectivity of the transformation. Notably, the incorporation of diols into the reaction mixture further enables the synthesis of poly(urea‐urethane) copolymers with *M*_n_ up to 2827 Da.

### 2.4 Poly(ester amide)s, Poly(urea urethane)s, and Poly(ester ether)s

**Scheme 18:**
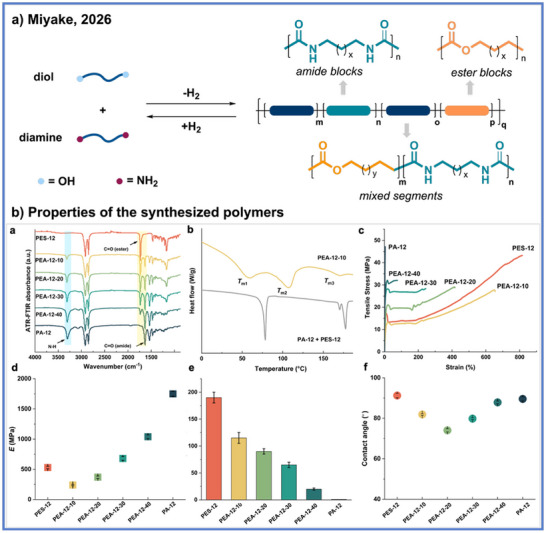
Synthesis of multi‐block poly(ester amide) materials via ADP from Miyake and coworkers. Copyright © 2026 Spring Nature.

Recent advances in ADP have expanded its application toward the synthesis of structurally complex multiblock copolymers. Building upon the earlier discovery of highly selective amide bond formation in polyamide synthesis via ADP, Miyake and coworkers developed a nucleophile‐governed, one‐pot sequential polymerization strategy to construct poly(ester amide) (PEA) multiblock architectures.^[^ ^64^ ^]^ Governed by monomer nucleophilicity and stoichiometry, they achieved the sequential formation of amide, gradient, and ester segments within a one‐pot polymerization, thereby enabling the synthesis of multiblock copolymers. Among various catalysts screened, the **Ru–I** complex exhibited the highest activity and selectivity for amide bond formation, effectively mediating the stepwise transformation of diamines and diols into multiblock PEA materials (Scheme 18a). The resulting materials integrate ester‐rich soft domains, which efficiently dissipate mechanical stress, with amide‐rich hard domains for structural reinforcement through dense hydrogen‐bonding networks. This hierarchical organization imparts a wide spectrum of mechanical performance (Scheme 18b) with Young's moduli ranging from 240 to 1040 MPa, tensile toughness up to 190 MJ m^−3^, and excellent thermal stability (*T*_d5_ > 360 °C). Furthermore, contact angle measurements and DSC/WAXD analyses confirmed the presence of well‐defined multiblock morphologies and tunable surface polarity.

**Scheme 19:**
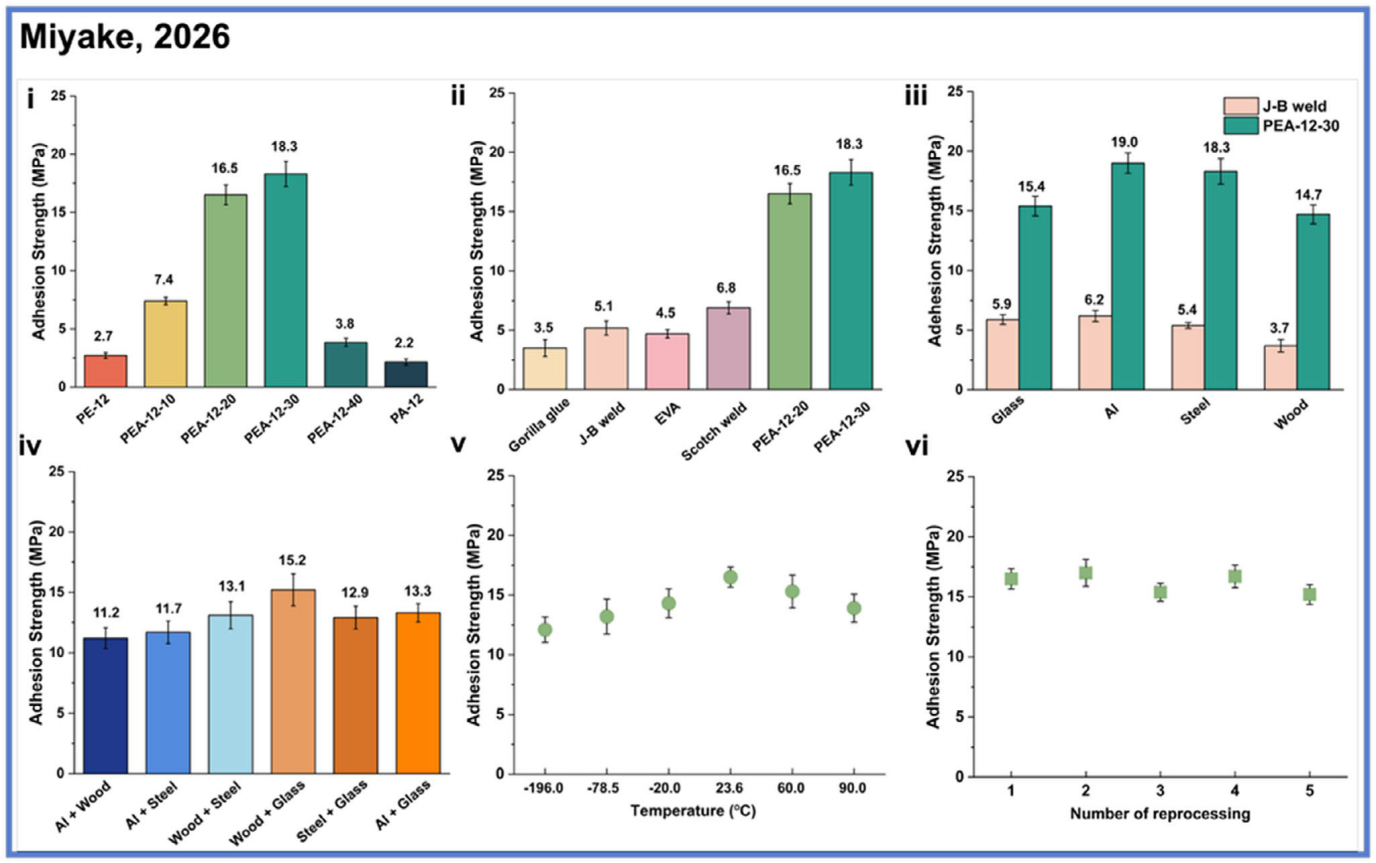
Evaluation of adhesion strength of multi‐block poly(ester amide) materials. Copyright © 2026 Spring Nature.

In addition to their tunable mechanical and thermal properties, these PEA materials exhibit promising functional performance as high‐strength adhesives (Scheme 19). When applied to steel substrates, they deliver lap‐shear strengths up to 19 MPa, exceeding those of commercial benchmarks, such as EVA and J‐B Weld, while maintaining excellent adhesion across a variety of other substrates (Scheme 19, i‐iv). Their bonding performance remains stable under extreme conditions such as exposure to temperatures ranging from−196 °C to 90 °C and harsh chemical environments, including acidic, basic, and organic solvent exposure (Scheme 19, v). Even after multiple debonding and rebonding cycles, the adhesives retain their mechanical integrity and cohesive strength, underscoring their durability and reprocessability (Scheme 19, vi). Equally important, these materials preserve their full closed‐loop chemical recyclability. Under catalytic hydrogenation with Ru‐, Mn‐, or Fe‐based systems, both diol and diamine monomers can be recovered in yields of up to 96%, even from complex mixtures containing commercial plastics, pigments, and stabilizers.

**Scheme 20:**
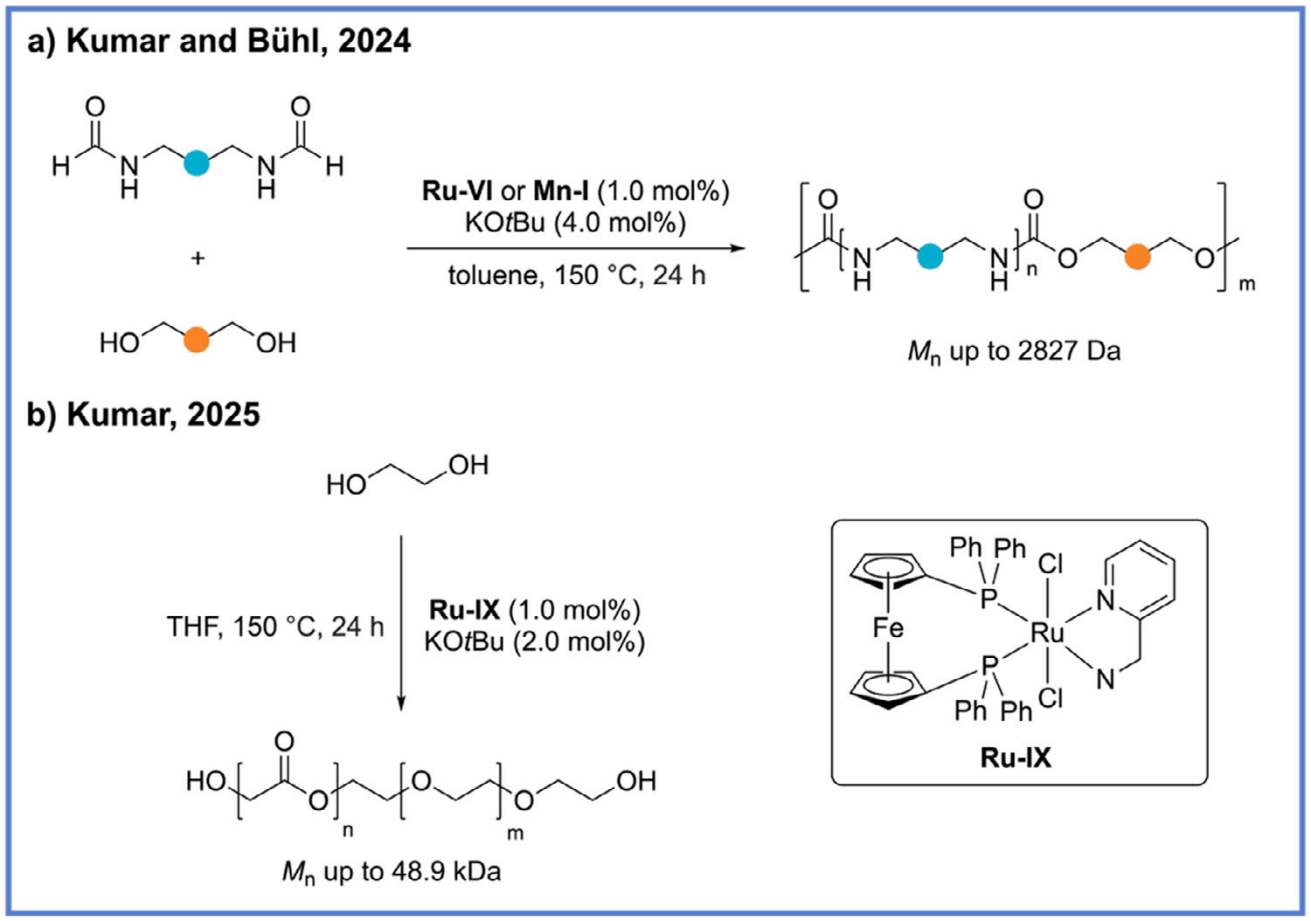
Synthesis of poly(ester ether) from EG via ADP from Kumar and coworkers.

Beyond the successful synthesis of poly(ester amide) materials, the ADP strategy has also been extended to the construction of other copolymers, such as poly(urea urethane)s and poly(ester ether)s. In 2024, Kumar, Bühl, and coworkers reported an elegant ADP approach employing diformamides and diols to access poly(urea urethane)s, in which both Ru‐ and Mn‐based catalysts were shown to be competent (Scheme 20a).^[^ ^63^ ^]^ This methodology enabled the formation of polymers with number‐average molecular weights of up to 2827 Da while proceeding in a fully atom‐economical manner with the concomitant liberation of H_2_ and CO as the sole byproducts. More recently, Kumar, Wolff, and coworkers reported a ruthenium‐catalyzed ADP strategy for the direct synthesis of poly(ester–ether) copolymers using EG as monomers (Scheme 20b).^[^ ^65^ ^]^ In this system, EG is first dehydrogenated to glycolaldehyde, which subsequently undergoes hemiacetal formation followed by intramolecular dehydration and further dehydrogenation, ultimately leading to the formation of alternating ester and ether linkages along the polymer backbone. Comprehensive DFT calculations and kinetic analyses elucidated the underlying mechanism, revealing that β‐hydride elimination from the Ru–alkoxide intermediate serves as the rate‐determining step, while the dynamic interplay between Ru–H and Ru–alkoxide species governs the chemo‐ and regioselectivity of ester versus ether bond formation. Interestingly, the polymer composition and connectivity could be systematically tuned by varying the chain length of the diol monomer, with short‐chain diols (e.g., ethylene glycol) favoring the formation of poly(ester–ether) copolymers with mixed linkages while long‐chain α,ω‐diols (e.g., 1,6‐hexanediol and 1,10‐decanediol) predominantly yield linear polyester structures.

## 3 Conclusions and Future Outlook

ADP represents a relatively new polymerization methodology for sustainable macromolecular synthesis by enabling the direct construction of polymers from simple, unactivated building blocks while generating molecular hydrogen as the only byproduct. Over the past decade, the field has evolved from conceptual proof‐of‐principle reactions to a versatile platform capable of producing diverse classes of polymers (such as polyesters, polyamides, polyurethanes, polyureas, etc.) with remarkable control over molecular architecture, recyclability, and performance. The integration of metal–ligand cooperative catalysis, green chemistry principles, and polymer design has firmly established ADP as a powerful bridge between homogeneous catalysis and polymer science. Looking ahead, several key directions will determine the trajectory and impact of ADP chemistry:

### 3.1 Catalyst Development

Despite significant progress, the catalytic landscape of ADP remains dominated by ruthenium‐based pincer complexes. Although emerging examples employing earth‐abundant metals such as manganese and iron demonstrate promising potential, their catalytic efficiencies and turnover frequencies are still limited, often requiring higher catalyst loadings or extended reaction times. The design of new ligand frameworks that enhance metal–ligand cooperativity, stability, and selectivity while maintaining cost‐effectiveness and scalability will be crucial to advancing practical ADP catalysis. Additionally, heterogeneous catalysts have been demonstrated in small molecule couplings and hold promise in polymer synthesis.^[^ ^66^, ^67^, ^68^ ^]^

### 3.2 Expansion of Application Scope

While ADP has been firmly established as a robust synthetic methodology, its translation into functional polymer applications remains nascent. Beyond recyclable plastics and high‐performance adhesives, future studies should leverage ADP to access advanced materials with targeted properties, such as self‐healing networks, stimuli‐responsive systems, and coatings. Coupling ADP with dynamic covalent chemistry, photochemical control, or flow‐based processes may further broaden its industrial and technological relevance.

### 3.3 Architectural Diversification

To date, most polymers synthesized via ADP exhibit relatively simple linear architectures. Expanding this chemistry toward branched, star, graft, and network polymers would unlock new opportunities in tuning mechanical, thermal, and interfacial properties.

Overall, continued advances in catalyst innovation, application‐driven material design, and structural diversification will elevate ADP from a promising synthetic concept to a general and scalable platform for next‐generation sustainable polymer chemistry.

## Conflict of Interests

The authors declare no conflicts of interest.

## Data Availability

Data sharing is not applicable to this article as no new data were created or analyzed in this study.
